# The meaning of learning to live with medically unexplained symptoms as narrated by patients in primary care: A phenomenological–hermeneutic study

**DOI:** 10.3402/qhw.v10.27191

**Published:** 2015-04-16

**Authors:** Eva Lidén, Elisabeth Björk-Brämberg, Staffan Svensson

**Affiliations:** 1 Institute of Health and Care Sciences, The Sahlgrenska Academy, University of Gothenburg, Gothenburg, Sweden; 2 Institute of Environmental Medicine, Karolinska Institutet, Solna, Sweden; 3 Angered Family Medicine Unit, Angered, Sweden

**Keywords:** MUS, primary care, person centred care, phenomenological-hermeneutics

## Abstract

**Background:**

Although research about medically unexplained symptoms (MUS) is extensive, problems still affect a large group of primary care patients. Most research seems to address the topic from a problem-oriented, medical perspective, and there is a lack of research addressing the topic from a perspective viewing the patient as a capable person with potential and resources to manage daily life. The aim of the present study is to describe and interpret the experiences of learning to live with MUS as narrated by patients in primary health-care settings.

**Methods:**

A phenomenological–hermeneutic method was used. Narrative interviews were performed with ten patients suffering from MUS aged 24–61 years. Data were analysed in three steps: naive reading, structural analysis, and comprehensive understanding.

**Findings:**

The findings revealed a learning process that is presented in two themes. The first, *feeling that the symptoms overwhelm life*, involved becoming restricted and dependent in daily life and losing the sense of self. The second, *gaining insights and moving on*, was based on subthemes describing the patients’ search for explanations, *learning to take care of oneself*, as well as *learning to accept and becoming mindful*. The findings were reflected against Antonovsky's theory of sense of coherence and Kelly's personal construct theory. Possibilities and obstacles, on an individual as well as a structural level, for promoting patients’ capacity and learning were illuminated.

**Conclusions:**

Patients suffering from MUS constantly engage in a reflective process involving reasoning about and interpretation of their symptoms. Their efforts to describe their symptoms to healthcare professionals are part of this reflection and search for meaning. The role of healthcare professionals in the interpretative process should be acknowledged as a conventional and necessary care activity.

Medically unexplained symptoms (MUS) is a condition that affects a large but heterogeneous group of people. The health services have so far been unsuccessful in addressing the healthcare needs of these people, partly because of outdated theories and diagnostic systems that fail to encompass the complexity of the patients’ health problems (Fink & Rosendal, 2008). The lack of a medical explanation and cure leaves patients and healthcare professionals in a situation where both parties may feel unsatisfied. However, recent research has shown promising results concerning the effectiveness of care methods such as mindfulness (Fjorback, Arendt, et al., 2013; Fjorback, Carstensen, et al., 2013) and stepped care (Gask, Dowrick, Salmon, Peters, & Morriss, 2011). In this study we focus on the patient's point of view by elucidating how persons suffering from MUS learn to live with the condition and strive to find meaning in a changed health and life situation.

The estimated prevalence of patients who seek primary care for MUS varies from 3 to 30% (Aamland, Malterud, & Werner, 2014; Kroenke, 2003; Wessely, Nimnuan, & Sharpe, 1999). The variation could be related to the different definitions of the condition but also to the range of inclusion criteria in research (Aamland et al., 2014; Barsky & Borus, 1999; Brown, 2007; Kroenke, 2003; Peveler, Kilkenny, & Kinmonth, 1997; Wessely et al., 1999). Extensive research has demonstrated the difficulty of explaining and managing MUS in healthcare settings (Barsky & Borus, 1999; Brown, 2007; Henningsen, Zipfel, & Herzog, 2007; Peveler et al., 1997; Rolfe, 2011; Salmon, Ring, Humphris, Davies, & Dowrick, 2009; Wessely et al., 1999) but the inexplicability of this condition is an unstable phenomenon, as medical explanations for the symptoms can sometimes be found (Gask et al., 2011; Leiknes, Finset, Moum, & Sandanger, 2006). This fact makes the healthcare situation unpredictable for patients as well as health professionals.

The causes of the patients’ symptoms have often been claimed to be of mental origin, as a consequence of their life situation and/or distress at deviation from cultural norms (Epstein, Quill, & McWhinney, 1999). In a literature review investigating psychological explanatory models, Rief and Broadbent (2007) identified a variety of cognitive, behavioural, and emotional aspects in research related to patients’ experiences and management of symptoms. Other studies emphasize that MUS is also associated with biological processes involving, for example, the endocrine and immune system, amino acids and neurotransmitters, as well as the persons’ physiological activation and cerebral activity. The combination of these psychobiological and psychological processes has been described as parts of a signal/filter system that affect the patients’ experiences of symptoms (Rief & Barsky, 2005). Rief and Broadbent (2007) concluded that existing models have strengths as well as weaknesses. Simplistic models (such as those that focus on attention, perception, and attribution) are too individualistic to explain the complexity of the origin of the symptoms. On the other hand, more developed models that include emotional, social, and interactional factors are primarily descriptive and lack explanatory power.

From the patient's point of view, living with MUS has been described as a struggle. Feelings of being in chaos, being a medical orphan, and that people perceive that the problems only exist in the mind have been described (Nettleton, Watt, O'Malley, & Duffey, 2005). Discriminating attitudes and categorization of people based on societal and normative prejudices have also been revealed in research: for example, a Danish study found that symptoms among patients with a low educational level were more frequently classified as psychological compared to highly educated patients, where the same symptoms were interpreted as tiredness due to a heavy work load (Mik-Meyer, 2011). Some patients with MUS reject psychological explanations for their problems as they consider them stigmatizing. Traditional methods such as reattribution that are solely based on medical and psychological science are therefore deemed too narrow to address the complexity of MUS, as the patients’ sociocultural context should also be considered when interpreting her or his symptoms (Gask et al., 2011). Nevertheless, many patients choose to adapt to the narrow medical framing and tacit demands of consultations in the healthcare context (Risør, 2009).

Research has emphasized the need for a personalized approach when caring for patients with MUS because of their heterogeneity (Aamland et al., 2014) and the importance of being attentive to patients’ emotions and interpretations of their health and life situation (Gask et al., 2011; Smith et al., 2006). However, in order for patients to be taken seriously, their narratives must be considered trustworthy by the listener (Hydén & Brockmeier, 2008; Werner & Malterud, 2003). If not, their request for care might be refused, leading to loss of moral esteem (Bülow, 2008). The patient's position in healthcare encounters is thus vulnerable and there is a risk that essential information about her or his health status and resources may be overlooked in the assessment and care planning process.

Although research about MUS is extensive, most studies seem to address the topic from a problem-oriented perspective. The absence of effective methods to relieve patients’ symptoms leads to the question of how healthcare professionals can support people with MUS to help them endure as well as manage the hardships in daily life.

In this study we have chosen to focus on the possibilities and resources for health and well-being as reported by those who suffer from MUS. We will therefore address the question of how people learn to live with MUS, despite a sometimes chaotic life situation and unpredictable future, and what they learn from their experiences. In line with, for example, Malterud et al. (2000), we see this knowledge as an expertise that should be acknowledged in healthcare encounters, education, and the development of care models. We have been inspired by a pedagogical perspective that highlights people's reflections as a means of developing new knowledge in situations where previous practices have proved unsatisfactory (Dewey, 2007; Schön, 2003). The perspective of health and healthcare that frames the study is person-centred, recognizing patients as whole persons and acknowledging their capacity and resources for health (Ekman et al., 2011; Smith, 2010). Our study is part of a project called Symptom Contextualization in Primary Health Care (SCPHC), comprising several studies with a variety of data collection methods. The aim of the present study was to describe and interpret the experiences of learning to live with MUS as narrated by patients in primary healthcare settings.

## Method

A phenomenological–hermeneutic method (Lindseth & Norberg, 2004) was used in this study. The participants were recruited between April and November 2011 at two suburban primary healthcare centres in Sweden. The inclusion criteria were as follows: age 18–64 years; at least eight visits to a physician or nurse at the healthcare centre during the previous 12 months; no specific organic or psychiatric cause for the frequent contact; and at least 50% of reported symptoms medically unexplained. These criteria are a modified version of those used by Smith et al. (2004).

**Figure 1:**
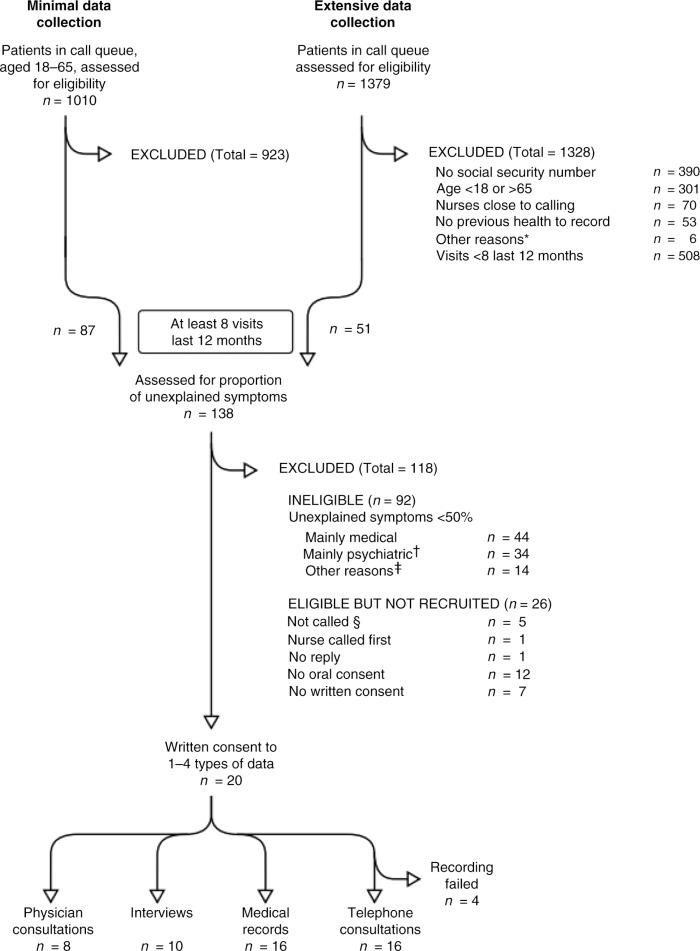
Flowchart of the data collection process.

Potential participants were identified by screening incoming telephone calls from patients to the healthcare centre (identification carried out by SS). Patients deemed suitable were later contacted by one member of the research team (EL or EBB), who asked them if they would be willing to participate in the study. The request was formulated in everyday language: “I am part of a research group investigating how patients with symptoms for which there is no specific medical explanation manage their daily life. Would you be willing to participate in such a study?” A total of 20 patients agreed to participate in one or more of the studies in the SCPHC project. Most participants were recruited during the extensive data collection period between September and November 2011 (see flow chart in Fig. 1). In the present study, ten individuals (three men and seven women) aged 24 to 61, seven of whom had an immigrant background, agreed to participate.

The time and place for the interview were chosen by the participants, who opted for their own home (5), the university (3), the healthcare centre (1), and a public library (1). The interviews, which lasted between 21 and 80 min (mean 59 min), were performed by EL and audio-recorded by means of a digital device. The researcher explained that she had not read the medical record nor had she any prior information about the participant. The opening question was, “Would you like to begin by telling me a little about yourself and your daily life?” Open-ended questions were posed about contextual factors such as work, healthcare, and family situation. In order to obtain a comprehensive narrative, the researcher encouraged the participants to link key events in a timeline. The audio-recorded interviews were transcribed verbatim by a professional secretarial service.

Interpretation of the data was carried out in three steps: naive reading, structural analysis, and comprehensive understanding (Lindseth & Norberg, 2004). The narratives were first read several times in order to grasp the essence of the text, which was formulated as the naive understanding. During the structural analysis a methodical and critical distance was maintained in order to validate or reject the naive understanding. In this step the intention was to describe “what the text said” (Lindseth & Norberg, 2004, p. 146) by remaining close to it and not taking the interpretation too far. The text was then taken out of context and divided into meaning units that were condensed, coded, compared, and finally categorized into subthemes and themes. Reflection on the naive understanding took place throughout the whole process. This reflection led to reconsideration of the naive understanding and a new structural analysis was carried out until agreement was achieved between the naive understanding and the result of the structural analysis. This process was mainly undertaken by the first author. Finally, the naive understanding and the structural analysis were theoretically and critically reflected upon in order to open into a new and deeper comprehension of “what the text was talking about” (Lindseth & Norberg, 2004, p. 146). In this step all authors contributed by applying their varying personal, professional, and scientific experiences to the text, thus ensuring that the interpretation was as critical and creative as possible.

### Ethical considerations

This study was approved by the regional Ethics Committee in Gothenburg (No. 115–11). Informed consent was obtained both orally and in writing from all participants.

## Findings

The participants’ narratives about learning to live with MUS concerned what they learned as well as how they learned. The narratives comprised their experiences, actions, and reflections from the day the symptoms started. The participants’ learning was interpreted as a process involving reflection about their previous life, the present, and the future.

### Naive understanding

Learning to live with MUS seems to be about coming to terms with shattered opportunities for ordinary daily life. The condition evokes feelings of being changed as a person and loss of joie de vivre. It involves reflection on how life used to be, including both bad and good memories, in addition to hope for as well as fear of the future. Learning to live with MUS is a struggle to interpret symptoms and manage a daily life that is dominated by them. The struggle sometimes leads to new insights about who you are and life per se.

### Themes

The structural analysis led to two themes and five subthemes (Table I) illustrating the learning process, which involved an increasing awareness about the body and bodily reactions, considerations about practical matters in daily life, and also reflections about existential issues. The first theme, *feeling that the symptoms overwhelm life*, describes how the participants recognized signs of illness and realized that a major change in life had occurred. The second theme, *gaining insights and moving on*, reveals how the participants approached the challenge of living with their inexplicable and unpredictable illness.

**Table I:** Overview of themes and subthemes

Theme	Subtheme
Feeling that the symptoms overwhelm life	Being restricted and dependent in daily life
	Losing the sense of self
Gaining insights and	Searching for explanations
moving on	Learning to take care of oneself
	Learning to accept and becoming mindful

#### Feeling that the symptoms overwhelm life

The participants described receiving signals from their bodies and sensing that something was wrong. They stated that the physical and psychological symptoms in combination with social problems more or less dominated life and described their difficulty accepting that they had become vulnerable and dependent. The narratives involved descriptions of a fight for control, structure, and safety in daily life.

#### Being restricted and dependent in daily life—

The participants described symptoms that caused physical immobility and inability to perform normal daily activities. They were unable to engage in recreational activities, keep their home in order, or maintain social relationships. Psychological symptoms such as anxiety also restricted daily life, leading to limitations that made the participants feel lonely. Even small disturbances in daily life could trigger excessive stress.

Pain and fatigue were two major reasons for restrictions in daily life. Pain could move all over the body and was described as slight or piercing with varying intensity. It could strike like a bolt of lightning and one participant described how she tried to “save herself” (P3). One participant claimed that she felt best when she was asleep, because then she was not tired and had no pain (P8), whereas another expressed that the body did not relax even when asleep (P6). Fatigue was described in terms of sleep deprivation, inability to rest, and a state of constant tension. The participants reported a paralyzing feeling of powerlessness, emptiness, lifelessness, and passivity. Circadian rhythm disturbances seriously affected family life. The participants also spoke about severe exhaustion leading to cognitive limitations: being unable to focus properly and the brain being “sluggish.” One woman described a feeling of being in a twilight zone:

P8: But always feeling tired is like living in a … [4-s pause] … twilight zone or something. I misunderstand what people say, I don't hear what they say, I can't follow and …

#### Losing the sense of self—

The participants’ inability to maintain their ordinary life and appearance evoked lack of self-confidence and feelings of lost identity and shame. The desire to work and earn money was emphasized, while shame about being unable to support oneself and one's family was expressed in the narratives. One participant said:

P7: “What kind of person am I?” I ask myself “Where do I get this money?” … I compare myself with my father.… He worked until he was 80 and doesn't approve if you don't work. So I lie and tell him that I am working.

For the participants from other countries, loss of language proficiency due to few contacts with native Swedish people during long-term sick leave was described as an obstacle when encountering healthcare professionals, causing feelings of powerlessness and of being illiterate (P2). The narratives involved descriptions of and reflections on how life had turned out and how the participants had changed. For example, one participant said that she did not recognize herself when she looked in the mirror: “The person I am today is a stranger” (P8).

#### Gaining insights and moving on

Over time the participants gained a distance that made it possible to detect patterns in relation to health and various life events. They searched for explanations that could make sense of their illness experience and developed strategies for managing a changed daily life. They used various methods to protect and strengthen their health. Learning to express symptoms and concerns was an important part of this process, which they practised when trying to describe their symptoms to physicians. Self-reflection and reflections with family members or friends were important for gaining perspective and finding new meaning in life.

#### Searching for explanations—

The participants used various explanatory frameworks when describing their impaired health, such as poor genetic immune defence (P7). Others interpreted their symptoms as being related to their personality: “Some can handle pressure, others can't” (P6). Their health and health situation were also described from a social perspective, as a result of difficulties involving lack of structure and control in private life and/or work, poor relationships, and traumatic events:

P5: … because at school I was always badly bullied. It was during my entire time at school so I was very depressed….

The participants’ work situation was described as related to their health; an overloaded work situation could be seen as a plausible cause of the symptoms:

P8: … But because I was constantly active and had kids and everything, I could never really reflect on … [3-s pause] … I was so busy that I never had time to stop and think at all.

EL: You just worked on?

P8: YES, I just worked on.

EL: Mm.

P8: And it is very likely … [4-s pause] … it is coming back at me now…

However, unemployment was also considered a cause of the illness:

P7: When you are unemployed, something happens to the body.

EL: Has unemployment really made you ill?

P7: Yes.

The narratives also contained sections where the participants clearly distinguished between “the illness” and “the person.” For example, one participant interpreted her depressed mood as a result of a tense body and not related to herself as a person (P6).

The participants wished that the healthcare professionals would listen to their experiences and discuss their interpretation of the symptoms. At times the opportunity to describe their experiences appeared to be more important than receiving a diagnosis:

P1: I have tried to explain [to the physicians] that I know there is a relationship between my psychological state [and the physical symptoms] but … I don't mean that they are not related but I feel different when I have this pain compared to when I only have psychological problems.

EL: What would you like them [the physicians] to do?

P1: I would like them to listen more instead of trying to put a name on it.

#### Learning to take care of oneself—

The narratives revealed that over time some participants learned to see new possibilities in life. Their ambition was to live in the “here and now” by avoiding all thoughts about the past and future. For example, one participant who suffered from constant pain described his efforts to ignore it while at the same time being cautious:

P4: I get even more … in other words, it becomes even worse so I have difficulty working with my hands, … a burning feeling and radiating pain.… I do things anyway, but I am more cautious.

The participants found strategies to cope with the symptoms and learned how to protect themselves, for example by shutting out destructive thoughts during dark days. Instead, they tried to take a detached view and attempted to maintain a positive attitude. One man described using a metaphor to promote a positive mindset:

P7: The stress management course taught me to see more clearly and … as my friend said, “You must always imagine that you are wearing a helmet. All these problems are like stones hitting your head, but if you wear a helmet they can't hurt you.”

The participants prioritized in order to function as “normally” as possible and feel well. Being able to work was described as important for well-being:

EL: What do you do to feel well?

P4: Well … the problem is … you know, working is actually very good for the body, you forget, you meet people, you feel good.

Rest was described as a prerequisite for the ability to work. It sometimes happened that they declined invitations to social events “because the weekends are reserved for rest”. (P5).

*Learning to take care of oneself* could also concern working in an ergonomically correct manner. The participants described how they tried to find ways to manage their body, regain control, and become strong. Some used meditation and yoga, while others found strength by taking walks in nature. Despite pain, most participants exercised regularly in various ways, which they considered necessary for functioning in daily life.

#### Learning to accept and becoming mindful—

The participants related the difficulty of coming to terms with their restricted health and life situation. It was described as difficult to be classified and to recognize oneself as a patient, especially if the participant in question had been on “the other side,” that is, a healthcare professional.

P6: Yes. You say to him [the physician] “… now I must learn but I feel weak. I believe that I must accept having to learn to live with my pain.” And although I have a lot of knowledge it does not help me emotionally.

In the narratives, relationships with other people were emphasized as a source of strength. Children and grandchildren strongly influenced the participants’ outlook on life; in some cases they were the only reason for living (P3, P7). Relationships with friends, animals, and God were significant for the participants’ well-being (P5). There were reflections on children's right to grow up in a loving family, as well as narratives about strengths, abilities, desires, and hope characterized by self-respect, pride, not being a victim, and a power that came from within. Some participants tried to visualize the future in a positive way, for example, being a good grandmother, while others were concerned about preventing the next generation from repeating their own or their parents’ mistakes. The narratives also involved reflections on life and insights that had developed over time. The participants talked about accepting a new and unfamiliar situation and realizing that not everything can be explained. The stories also contained conclusions that, despite the suffering, life had also contained joy:

P1: But at the same time, there has also been joy in life. If it had only been misfortune it would have been different, but I have had both.

Moreover, there were statements that highlighted the value of their experiences, not only for themselves but for others:

P5: But later, when you have got over the worst and you can see how you came through, you can help others by sharing your experiences.

### Comprehensive understanding

The meaning of learning to live with MUS was interpreted as a great and difficult challenge that involves losing control over life, becoming disoriented, and then beginning a battle to reorient. This battle involves making sense of the body and life per se, an endeavour that encompasses both a practical level, functioning in daily life, and an existential level, discovering new and sometimes unexpected aspects of oneself. The narratives indicated variation in the participants’ trust in their personal abilities. Their experience of learning to live with the symptoms had different outcomes; some believed that their basic self-image was preserved and even strengthened, whereas others felt so different they no longer recognized themselves.

We have chosen to interpret the results of the naive reading and structural analysis by means of Kelly's personal construct theory (PCT) in combination with Antonovsky's theory of sense of coherence (SOC) (Antonovsky, 1987; Butt, 2008). According to the PCT, people can be seen as “scientists” who continuously search for ways to find meaning in life by testing hypotheses and methods to develop an expedient and sustainable personal interpretative framework, that is, a system of constructs (Butt, 2008). These are described as fundamental but not deeply considered personal views about issues such as self-image, relationships, and life circumstances. Individuals’ repertoire of constructs is not static but developed in a dynamic, ongoing process, which allows for adjustment if they become inadequate in times of change (Butt, 2008). The use of SOC in this interpretation was seen as appropriate because of its comprehensive approach integrating the human dimensions of cognition, activity, and existentiality in the process of people's meaning-making in addition to its salutogenic foundation (Antonovsky, 1987).

The first theme, *feeling that the symptoms overwhelm life*, illuminates the sense of confusion, fear, frustration, and strangeness that emerges when the symptoms start to appear. The participants tried to protect themselves from the new and unfamiliar bodily sensations, which raised questions about what was going on. They began to reflect on and question assumptions previously taken for granted: truths about themselves as people, their relationships, and circumstances in their sociocultural context. This reflection highlighted the need for new interpretative tools as, according to Butt, Kelly would have described it (Butt, 2008).

The second theme, *gaining insights and moving on*, concerns how the participants struggled to reconstruct their life. This process can be illustrated by Antonovsky's theory of SOC. The subtheme *searching for explanations* is related to comprehensibility; *learning to take care of oneself* deals with manageability; and *learning to accept and becoming mindful* concerns the search for meaning. Comprehensibility refers to how a person perceives a stimulus as rational, graspable, and clear (Antonovsky, 1987). In this study, the findings demonstrated that the participants’ search for explanations involved a wide range of psychological, social, cultural, and organizational perspectives. The participants were eager to discuss their personal interpretations with healthcare professionals as well as with friends and family members. Even if they were unable to agree about the interpretation, they welcomed opportunities to formulate their experiences and thoughts and obtain serious feedback on their reflections.

Regarding manageability, we found that the participants used a variety of health resources, which is similar to what Kelly described as “positive projects” (Butt, 2008). Concrete actions such as exercising, looking after pets, nurturing relationships with family and friends, and spending time in nature were described as increasing their energy level, as well as maintaining and enhancing a positive self-image of being physically active, strong, independent, responsible, and competent. In other cases the participants performed cognitive exercises in order to learn how to recognize the opportunities in life, live in the here and now, and prevent destructive thoughts. Some stated that they had stepped out of their ordinary context and had learned to manage their symptoms by keeping a distance, which enabled them to identify new and more appropriate constructs for their changed health and life situation. For example, a reconstructed and more tolerant self-image could emerge when a person managed to preserve a sense of being valuable and worthy despite vulnerability and dependency.

Some of the participants in our study related that they actually reached a point where they found new meaning in life. Their changed situation had forced them to reconsider core values and reflect on their future personal and professional life. Values related to physical functioning, love, and close relationships were problematized when considering existential questions such as “What kind of person am I?” and “Who do I want to be?” Some participants used their parents as positive role models, while others considered their parents poor examples best avoided. They mentioned significant others, friends, and family members (especially children and grandchildren) as important for achieving distance, viewing problems from a different perspective, and finding meaning.

## Reflections

The findings reveal that the participants were capable of giving a narrative about health resources and how they had developed strategies for managing daily life and making sense of their health situation. Several narratives contained deep reflections on health and life. Their efforts to find meaning were illustrated in their narratives of trying to verbally express, to themselves and others, how they experienced their bodily sensations and how these affected them as people. This is in line with Bruner's view of narration as a fundamental form of human communication by which people can organize and make sense of their experiences (Bruner, 1986). In that sense, narration per se can be seen as therapeutic. However, being in the middle of an illness process makes it difficult for the suffering person to apprehend and formulate their experiences in a comprehensive way to themselves or others. Instead, complex illness narratives tend to become fragmented and “broken” (Hydén & Brockmeier, 2008). Bülow (2008) reported that patients want healthcare professionals to assist them in their storytelling by asking questions. Healthcare professionals could thus facilitate the cocreation of a more coherent and meaningful story, a point which is also emphasized in other studies (Bülow, 2008; Dwamena, Lyles, Frankel, & Smith, 2009). According to our findings, such joint storytelling could involve conversations about how to gain distance to suffering in daily life, how to protect oneself, sources of well-being, and visualizing the future. From a clinical point of view, this is also in line with Stone (2013), who suggested five strategies to be employed in clinical encounters with patients suffering from MUS: acknowledging the patient's suffering and assuming responsibility as a carer; tolerating the uncertainty of the situation; acknowledging the desire to name the symptoms and obtain a cure; shifting the focus from cure to coping; and giving the patient unconditional positive support and/or finding a story that justifies suffering.

Barriers to such assistance can be found on several levels. On the individual level, research has shown that patients as well as healthcare professionals may be reluctant to elaborate on all aspects of MUS in healthcare encounters; for example, psychological issues can be experienced as threatening by both parties (Hilbert, Martin, Zech, Rauh, & Rief, 2010; Peters et al., 2009; Stone, 2013). Counselling could thus be required for healthcare professionals. At a structural level, as a consequence of the New Public Management movement, the demand for effectiveness involving economic incentives and time restrictions on consultations has had an impact on the treatment of patients in healthcare as well as in insurance organizations. Cost-effectiveness has been prioritized over patient education; due to a heavy workload and lack of time, care managers claim that they cannot prioritize patient education provided by nurses, nor the development of nurses’ pedagogical competence (Bergh, Friberg, Persson, & Dahlborg-Lyckhage, 2014).

Finally, we would like to challenge the dominant, one-sided medical perspective in care organizations for cases where no cure is available (Stone, 2013). Physicians are not necessarily the most appropriate actors and the consultation might not be the best arena in which to support patients with MUS in their learning process. A more comprehensive view of health and healthcare both in society and healthcare organizations could open the way for other care models and actors (e.g., nurses, health educators, and/or peer patients) that could contribute to a more needs-driven and sustainable care. One way to enable peer learning is by creating professionally organized and structured patient groups where patients with similar experiences are free to express and reflect on their experiences together. As an interesting example, Hinrichsen (2012) recommended a model for clinical practice (not yet evidence-based) involving a group of five to eight patients assessed as eligible for such an activity. In addition, the group should include one “expert patient” who has reached a certain level in her or his mastery process and one healthcare professional for support. We suggest that these person-centred models, which actually occur in clinical practice, should be compared with traditional care in well-designed research studies in order to obtain evidence of appropriate methods of caring for patients with MUS.

The credibility of the present study was strengthened by the fact that the participants were selected by the research team, whose members had no previous relationship with the patients. The data collection was performed by a researcher who was not involved in any professional documentation about the patient, the time and place for the interviews were chosen by the participants, and the data analysis was carried out in accordance with a previously used, systematic, and structured method (Iranmanesh, Ghazanfari, Sävenstedt, & Häggström, 2011; Johansson, Bergbom, & Lindahl, 2012; Martinsson, Fagerberg, Lindholm, & Wiklund-Gustin, 2012). The three steps of the analysis involved a critical and questioning approach in combination with detailed documentation of the process, which ensured internal validation (Whittemore, Chase, & Mandle, 2001). However, the study also has a limitation in that many patients declined participation. This point leads to the question of what motivated our participants to take part in the study and whether they differed in some respects from others who suffer from MUS. However, according to the inclusion criteria, which are consistent with an internationally recognized classification, they do not differ in any way. A strength of the study is that the findings highlight an important aspect that is often missing in this area, namely the patient's capacity.

## Conclusions

Living with MUS means struggling to find plausible explanations for one's suffering. The participants in this study demonstrated an ability to identify positive projects in life and things that made them feel good. They constantly engaged in a reflective process involving reasoning about and interpretation of their symptoms. Their efforts to express unexplained symptoms to healthcare professionals are part of their reflection and meaning-making. Healthcare professionals could facilitate the interpretative process by including MUS patients and entering into dialogue with them, which should be acknowledged as a necessary and conventional care activity.
